# Delayed Tracheostomy in a Patient With Prolonged Invasive Mechanical Ventilation due to COVID-19

**DOI:** 10.7759/cureus.8644

**Published:** 2020-06-15

**Authors:** Ian C Holmen, Andrew Kent, Stephanie Lakritz, Claire Brickson, Katarzyna Mastalerz

**Affiliations:** 1 Anesthesiology, University of Colorado, Denver, USA; 2 Internal Medicine, University of Colorado, Denver, USA; 3 Internal Medicine, University of Colorado Anschutz Medical Campus, Denver, USA; 4 Hospital Medicine, Eastern Colorado Veterans Affairs Medical Center, Aurora, USA; 5 Hospital Medicine, University of Colorado, Aurora, USA

**Keywords:** endotracheal intubation, tracheostomy placement, acute respiratory distress syndrome

## Abstract

Coronavirus disease 2019 (COVID-19) can cause acute respiratory distress syndrome (ARDS) that is associated with high mortality among patients requiring invasive mechanical ventilation. We present a case of a 56-year-old male with hypertension and obesity who presented with chest pain from COVID-19. The patient required endotracheal intubation due to worsening hypoxia and remained intubated for 33 days. Tracheostomy placement was delayed in part due to persistent COVID-19 positive testing until hospital day 37. The patient required a total of 52 days in the ICU prior to discharge to a rehabilitation facility. This case highlights the extensive resources needed for critically ill patients with COVID-19 and the long duration that patients may test positive for the virus after onset of symptoms. It also raises questions about the timing and safety of tracheostomy placement among those patients requiring mechanical ventilation from COVID-19.

## Introduction

Coronavirus disease 2019 (COVID-19) causes acute respiratory distress syndrome (ARDS) [1]. In a retrospective review from Wuhan, China, 26% of patients hospitalized for COVID-19 required ICU admission due to ARDS [2]. Twelve percent of patients in the New York City area admitted for COVID-19 needed endotracheal intubation, and mortality for those requiring invasive mechanical ventilation reached as high as 88.1% [3]. While there have been significant data on the mortality of patients requiring mechanical ventilation no previous case reports have detailed the clinical timeline of respiratory recovery in those requiring tracheostomy placement.

## Case presentation

**Figure 1:**
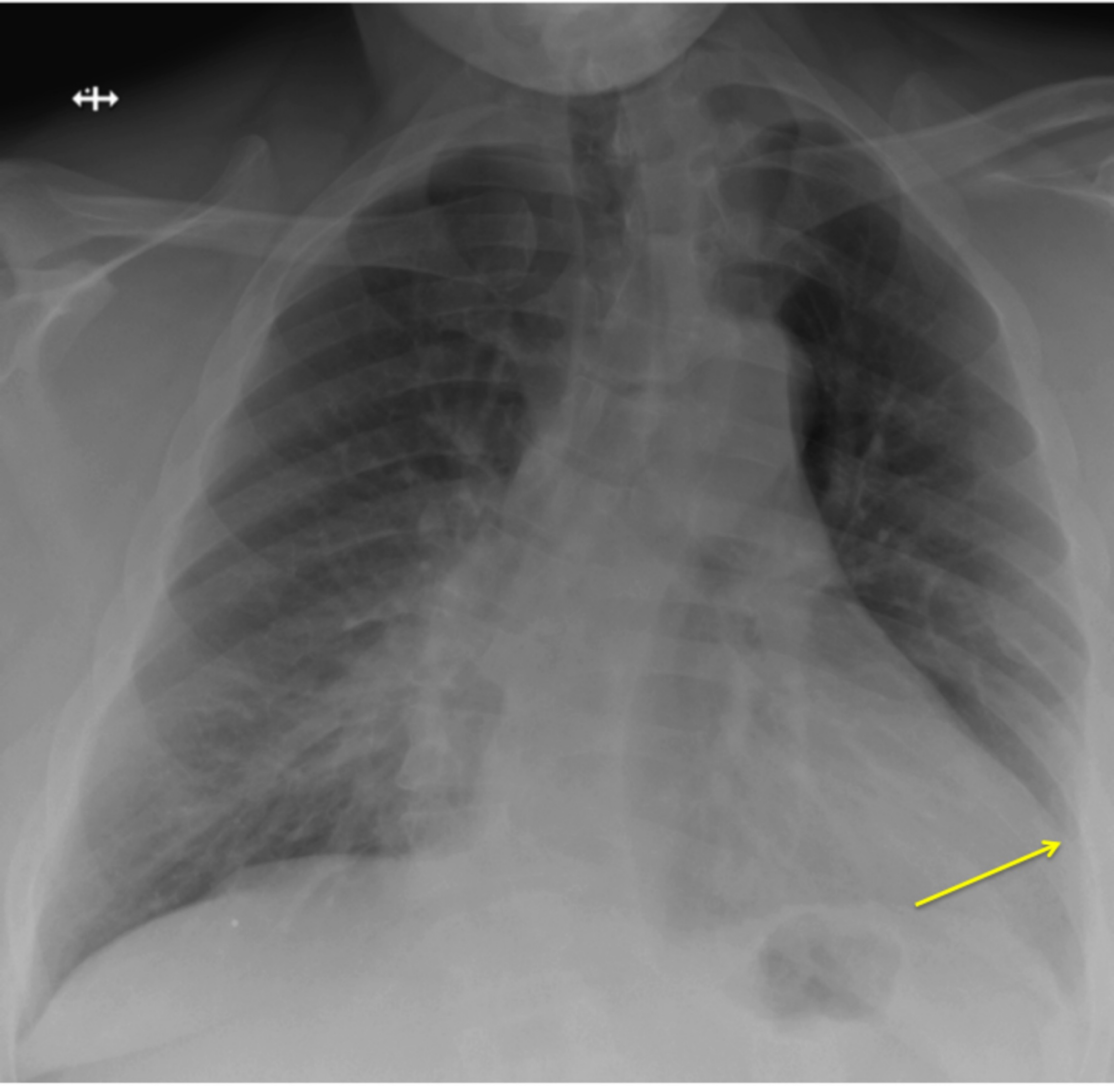
Chest X-ray on admission revealed normal lung findings and cardiomegaly. Yellow arrow indicating left lateral boarder of enlarged cardiac silhouette.

A 56-year-old male with obesity, hypertension, and scoliosis presented to the hospital for dull, atypical chest pain. He was febrile to 38.0 degrees Celsius and had a new oxygen requirement of two liters on nasal cannula for a blood oxygen saturation (SpO_2_) of 96%. His HEART score was four [4]. His initial D-dimer was 790 ng/mL, and his initial troponin in the ED was 0.077 ng/mL (normal less than 0.015 ng/mL), without evidence of dynamic changes on electrocardiogram (EKG). A respiratory viral panel was negative. CT angiography scan showed no signs of pulmonary embolism, and chest X-ray demonstrated normal lung findings without definitive acute infiltrates (Figure 1). The patient was admitted for hypoxia and acute coronary syndrome rule out. Repeat troponin level at six hours after admission decreased to 0.06 ng/mL. Given the patient’s new hypoxia and chest pain, a COVID-19 nasal swab was collected.

**Figure 2:**
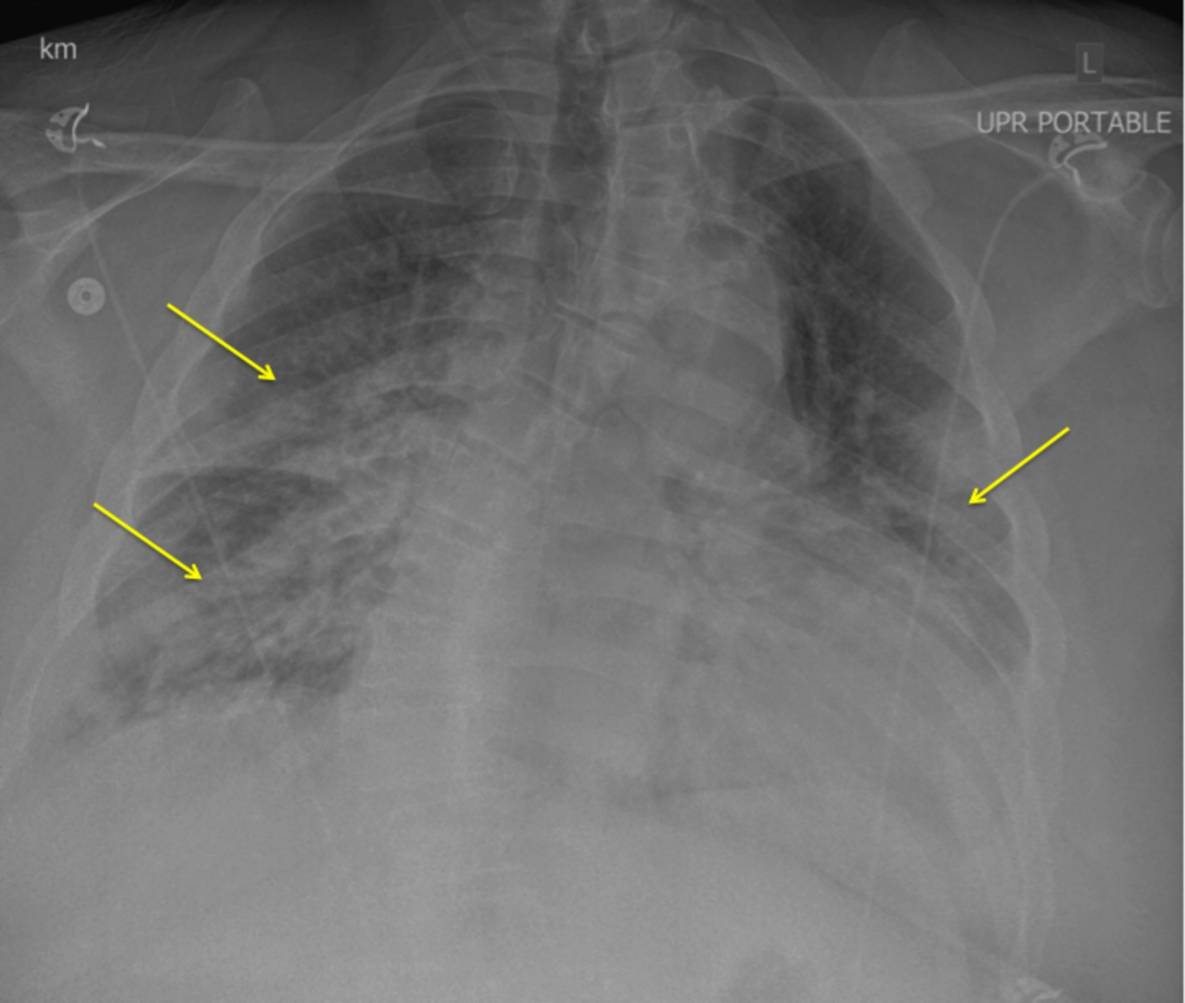
Chest X-ray (day 5) revealed bilateral infiltrates. Yellow arrows demonstrate increased opacity bilaterally.

On hospital day three, bilateral crackles on exam were presumed to be pulmonary edema, and he was diuresed with IV furosemide. However, diuresis was discontinued due to a subsequent acute kidney injury. On hospital day four, COVID-19 polymerase chain reaction (PCR) resulted positive, and the patient was started on a 10-day course of hydroxychloroquine. On hospital day five, the patient had worsening tachypnea and SpO2 was 78% despite six liters oxygen via nasal cannula. The patient consented for invasive mechanical ventilation. Immediately following endotracheal intubation, arterial blood gas demonstrated a ratio of arterial oxygen partial pressure to fractional inspired oxygen (P/F ratio) of 77. Repeat chest X-ray showed bilateral diffuse infiltrates consistent with severe ARDS (Figure 2). Over the next two days, the patient was placed in the prone position for 18 hours a day and was intermittently paralyzed with cisatracurium. Positive end-expiratory pressure (PEEP) was initiated at 20 cmH_2_O with 6 mL/kg low tidal volume ventilation. Plateau pressures remained below 30 cmH_2_0 with at this level of PEEP.

The patient continued to require mechanical ventilation with an endotracheal tube for the next 27 days. After the two days of intermittent prone positioning, oxygen saturation improved gradually over 14 days. The P/F ratio stabilized at greater than 200 on a PEEP of 5-7 cmH_2_O with FiO_2_ at 40%. Despite improvement in respiratory status, impaired mental status precluded extubation for several weeks. On hospital day 32, he was extubated to nonrebreather mask with 10 L oxygen supplementation.

Four days following extubation, on hospital day 36, he required re-intubation for hypercarbic respiratory failure. The patient had negative COVID-19 nasopharyngeal testing for the first time on hospital day 37, and consequently was scheduled for tracheostomy placement on hospital day 41. After tracheostomy, the patient had continued need for PEEP of 5 cm H_2_O and supplemental oxygen. The patient was completely weaned from pressure support on hospital day 55, 52 of which were in the ICU. He was discharged to a rehabilitation center after hospital day 58.

## Discussion

We present a case of COVID-19 requiring prolonged endotracheal intubation with mechanical ventilation for 33 days. The patient ultimately required tracheostomy placement prior to recovery and discharge. Literature to date has demonstrated high mortality among patients requiring invasive mechanical ventilation [3, 5]. Little information has been published regarding the course of recovery and timing of tracheostomy in COVID-19 case reports.

Prior reports from Wuhan, China suggested the median ICU length of stay for patients with COVID-19 was 8 days [2]. Here we report a 58-day hospitalization requiring 52 days in the ICU due to continued need for mechanical ventilation. It is also noteworthy that the patient continued to test positive for COVID-19 until 37 days after admission. Prior research suggests that viral shedding after symptom onset gradually decreases toward undetectable levels around day 21, which is significantly shorter than what we report [6]. While this case may demonstrate an outlier regarding time to negative COVID-19 testing in a patient with severe ARDS, some patients may still be infectious beyond the typical three-week period. It also demonstrates the level of intensive healthcare resources that may be needed to allow patients to recover.

To our knowledge, this is the longest course of endotracheal intubation and mechanical ventilation with survival that has been reported in the COVID-19 literature. Prior research in patients with pneumonia requiring mechanical ventilation suggests that tracheostomy placement within seven days of endotracheal intubation is associated with improved mortality, shorter ICU stays, and fewer days of mechanical ventilation compared tracheostomy placement between 14 and 21 days [7-8]. In this case, tracheostomy placement was delayed due to persistently positive COVID-19 PCR coupled with the possibility of successful extubation given the patient's improving respiratory status. In order to improve individual patient outcomes, and to better optimize ICU capacity during this pandemic, earlier tracheostomy placement could be considered. However, the benefits of early tracheostomy need to be balanced with the risk of aerosol-generating procedures and healthcare worker risk of infection [9].

## Conclusions

This case of COVID-19 infection illustrates prolonged endotracheal intubation, ICU stay, and viral shedding in a patient who ultimately recovered. The clinical trajectory may help inform decision making in similar COVID-19 infection cases in the future. Furthermore, this case raises important clinical questions regarding timing of tracheostomy placement. Decisions on timing may impact ICU capacity during the course of the COVID-19 pandemic given the prolonged hospital stays in critically ill patients.
